# Feasibility of anticoagulation-free peripheral veno-arterial extracorporeal membrane oxygenation in re-do lung transplantation

**DOI:** 10.1007/s10047-025-01541-8

**Published:** 2025-12-15

**Authors:** Chitaru Kurihara, Yudai Miyashita, Taisuke Kaiho, Dai Yamanouchi

**Affiliations:** 1 Department of Surgery, Northwestern University Feinberg School of Medicine, 676 N. St Clair St, Suite 650, Chicago, IL 60611 USA; 2 Department of Vascular Surgery, Fujita Health University, Toyooke, Japan; 3 Division of Thoracic Surgery, Northwestern University Feinberg School of Medicine, 676 N. Saint Clair St., Suite 650, Chicago, IL 60611 USA; 4 Division of Thoracic Surgery, Department of Surgery, Northwestern University Feinberg School of Medicine, 676 N. Saint Clair St., Suite 650, Chicago, IL 60611 USA

**Keywords:** Lung retransplantation, Heparin-free extracorporeal membrane oxygenation, Peripheral venoarterial ECMO, Blood transfusion requirement, Primary graft dysfunction

## Abstract

**Background:**

To evaluate the feasibility and safety of anticoagulation-free peripheral veno-arterial extracorporeal membrane oxygenation (VA-ECMO) during lung re-transplantation and to assess its impact on blood transfusion requirements and clinical outcomes.

**Methods:**

In this single-center retrospective cohort (January 2023–April 2025), we included adults undergoing bilateral re-do lung transplantation on peripheral VA-ECMO with an anticoagulation-avoidance protocol; primary lung transplants were not included. Data on patient demographics, intraoperative transfusion volumes, postoperative complications, and survival were collected. The primary outcomes were intraoperative packed red blood cell transfusion volume and overall survival; secondary outcomes included incidence of primary graft dysfunction, acute kidney injury, and hemorrhagic and thromboembolic events. Continuous variables are reported as medians with interquartile ranges, and survival was estimated using the Kaplan–Meier method.

**Results:**

Seven patients (median age, 42 years; range, 30–56 years) underwent re-transplantation for chronic lung allograft dysfunction. The median intraoperative transfusion requirement was 560 ml (interquartile range 280–1050 ml). One patient developed primary graft dysfunction of grade 3and two developed stage 3 acute kidney injury requiring renal replacement therapy. Two developed deep venous thrombosis nonrelated to ECMO cannulation; no pulmonary embolism occurred. At a median follow-up of 469 days, all patients survived without evidence of recurrence of chronic lung allograft dysfunction.

**Conclusions:**

Full anticoagulation-free peripheral VA-ECMO during lung re-transplantation is feasible and safe, with acceptable complication rates and potential reduction in transfusion requirements. Larger, multicenter studies are warranted to confirm these findings.

## Introduction

Lung transplantation (LTx) is a critical treatment for patients with end-stage pulmonary diseases, such as chronic obstructive pulmonary disease (COPD) and pulmonary fibrosis [1–3]. However, it is associated with perioperative challenges, especially in patients with cardiopulmonary failure. Recently, veno-arterial extracorporeal membrane oxygenation (VA-ECMO) is commonly used to provide mechanical circulatory support during LTx, ensuring adequate tissue perfusion and supporting cardiac and respiratory function during [4]. Despite its benefits, VA-ECMO poses significant complications, notably bleeding and thrombotic events. Heparin, typically used to prevent thrombus formation, increases blood loss, transfusion requirements, and bleeding-related complications. Up to 50% of lung transplant recipients experience significant blood loss, often requiring more than 10 units of blood products, which can contribute to poorer outcomes like acute kidney injury (AKI) and prolonged hospital stays [5]. This has led to growing interest in full dose heparin-free VA-ECMO protocols, which may mitigate bleeding risks and reduce transfusions while maintaining circulatory support. Several studies have investigated full dose heparin-free VA-ECMO in transplant settings, showing that it may reduce bleeding and transfusion needs without compromising outcomes [6–9]. While most studies focus on central VA-ECMO, peripheral VA-ECMO—using femoral cannulation—offers distinct advantages, such as fewer insertions and easier placement when re-do lung transplant, yet its effectiveness without heparin is still unclear. The feasibility and outcomes of full dose heparin-free peripheral VA-ECMO in lung transplantation need further investigation. Existing studies focus on central VA-ECMO [10–12] or non-lung transplant populations [13–15], leaving a gap in knowledge regarding full dose heparin-free management in lung transplant recipients. It is uncertain whether this approach can offer equivalent survival rates and clinical outcomes while reducing bleeding and transfusion requirements compared to traditional protocols. Additionally, the impact on pulmonary complications, graft survival, and kidney function remains unclear. We specifically focused on bilateral redo lung transplantation, a niche subgroup characterized by higher bleeding and transfusion risk. This clinical context provides the rationale for evaluating full anticoagulation-avoidance during peripheral VA-ECMO. This study aims to evaluate the feasibility and safety of full anticoagulation-free peripheral VA-ECMO during lung transplantation, contextualized to prior central VA-ECMO series. Specifically, we sought to assess whether this approach reduces blood loss, transfusion requirements, and provides similar clinical outcomes compared to those previously reported for central VA-ECMO.

## Materials and methods

### Study design

The study was approved by the Institutional Review Board of Northwestern University (STU00207250 and STU00213616). The need for patient consent for data collection was waived by the institutional review board due to the retrospective nature of this study. Patient data were collected retrospectively using electronic medical records and stored in a database at the Northwestern University Medical Center in Chicago, Illinois, USA. We included only consecutive adult recipients of bilateral redo lung transplantation between January 2023 and April 2025 who were supported intraoperatively with peripheral VA-ECMO without full anticoagulation. Multiorgan transplants were excluded. Data on patient demographics, comorbidities, donor characteristics, preoperative laboratory values, intraoperative and postoperative outcomes were collected. Early post-transplant complications, and survival outcomes were assessed. For contextual benchmarking, we also abstracted a contemporaneous cohort of intraoperative central VA-ECMO cases without full anticoagulation performed during the same interval (*n* = 308). Multiorgan transplants (*n* = 13) were excluded from all descriptive summaries. Across 510 single-organ lung transplants, VA-ECMO was used intraoperatively in 308 cases (60.4%). Because our re-do program adopted the heparin-free peripheral approach from inception, we did not have pre-protocol institutional re-do comparators.

## Intraoperative peripheral VA-ECMO management during lung transplant

Our intraoperative peripheral VA-ECMO management during lung transplantation has been described previously [16, 17]. In brief, once allograft arrived at the operative room, the femoral artery was exposed and was cannulated using an 16 F Fem-Flex cannula, and the femoral vein was cannulated using a 25-French Bio-Medicus cannula, and then the patients were placed on VA-ECMO. We administered 5,000 U of unfractionated heparin before cannulation. Patients were not monitored with bleeding parameters such as ACT or aPTT. The VA-ECMO was initiated and blood flows maintained at > 2.5 L/min. The VA-ECMO circuit included Quadrox iD adult (7.0) oxygenator (MAQUET Holding B.V. & Co. KG, Germany) and Rotaflow pump (MAQUET Holding B.V. & Co. KG, Germany). The cannulas were not coated with heparin, however, components of the circuit including the tubing and oxygenator were. Oxygen saturations were monitored on the right hand to ensure adequate oxygenation of arch vessels. After allograft was implanted, pump flows were maintained with pulse pressure above 10 mmhg on the arterial line to obtain right ventricular cardiac output of 1.5–2 L/min, which can prevent thrombus in the allograft [13–15]. The arterial and venous cannulas were removed after establishing hemodynamic and respiratory stability. I In our program, intraoperative heparin-free peripheral VA-ECMO is confined to short, planned runs during lung transplantation. Based on our experience and prior reports, we consider this approach safe within the intraoperative window; therefore, we do not maintain a dedicated protocol specifying numeric duration limits or stepwise “rescue” measures for exceeding a predefined threshold.

## Definition of complication

### Primary graft dysfunction (PGD)

PGD was defined based on the ISHLT guideline and graded by PaO2/FiO2 ratio as follows; Grade 1: PaO2/FiO2 ratio > 300; Grade 2: PaO2/FiO2 ratio is 200–300; Grade 3: PaO2/FiO2 ratio < 200. The use of ECMO for bilateral pulmonary edema on chest X-ray was classified grade 3 [18].

## Chronic lung allograft dysfunction (CLAD)

CLAD is defined as a sustained (at least 3 months) decline in the forced expiratory volume in one second (FEV₁) of at least 20% from the post-transplant baseline, in the absence of other reversible causes. CLAD is further sub-classified into phenotypes such as bronchiolitis obliterans syndrome (BOS) and restrictive allograft syndrome (RAS) based on clinical, radiologic, and physiologic criteria [19].

### Acute kidney injury (AKI)

AKI was defined using the Risk, Failure, Loss of kidney function, and End-stage kidney disease classification [20].

### Statistical analysis

Continuous variables are summarized as median [IQR] and categorical variables as n (%). Where between-group comparisons are shown, they were performed using the Wilcoxon rank-sum test for continuous variables and Fisher’s exact test for categorical variables. Overall survival was displayed using Kaplan–Meier curves and log-rank tests, with follow-up truncated at 1,000 days. Analyses were conducted in R (v4.5.1) using base functions and the packages survival and survminer.

## Results

### Patient characteristics

This cohort comprised seven patients who underwent bilateral redo lung transplantation (Table 1). Their ages ranged from 30 to 56 years, with six men and one woman. Body mass index values varied between 20.4 and 31.2 kg/m², while body surface area fell between 1.57 and 2.00 m². Only one patient reported a history of cigarette smoking. Hypertension and diabetes mellitus were each present in four and three patients, and chronic kidney disease in two. Allocation priority was assigned according to the Lung Allocation Score in one patient (LAS = 40.5) and the Composite Allocation Score in the remaining ssix (CAS range, 23.8–42.0). All seven recipients were retransplanted specifically for CLAD/BOS (chronic lung allograft dysfunction / bronchiolitis obliterans syndrome).

**Table 1:** Patients Characteristics

Case	Age	Gender	BMI	BSA	Single/ Bilateral	Smoking	HTN	DM	CKD	LAS	CAS	Etiology of Lung failure	Day from initial transplant to redo	
1	44	Male	22.7	1.65	Bilateral	No	No	No	No	40.5	-	CLAD/BOS	1807	
2	55	Male	20.6	1.57	Bilateral	No	Yes	Yes	No	-	42.0	CLAD/BOS	481	
3	30	Male	21.2	1.82	Bilateral	No	No	No	No	-	38.4	CLAD/BOS	854	
4	38	Male	31.2	1.80	Bilateral	No	No	No	No	-	27.1	CLAD/BOS	8165	
5	40	Male	20.4	1.90	Bilateral	Yes	Yes	Yes	Yes	-	23.8	CLAD/BOS	2552	
6	42	Female	24.0	1.83	Bilateral	No	Yes	No	Yes	-	25.6	CLAD/BOS	255	
7	56	Male	23.4	2.00	Bilateral	No	Yes	Yes	No	-	36.7	CLAD/BOS	1591	

BMI, body mass index; BSA, body surface area; HTN, hypertension; DM, diabetes mellitus; CKD, chronic kidney disease; LAS, lung allocation score; CAS, composite allocation score; CLAD, chronic lung allograft dysfunction; BOS, bronchiolitis obliterans syndrome.

### Baseline laboratory and arterial blood gas findings

At the time of transplantation, hemoglobin values ranged from 7.3 to 10.8 g/dL, with white blood cell counts spanning 2.7 to 14.0 × 10³/mm³ and platelet counts between 119 and 388 × 10³/mm³ (Table 2). Serum sodium was uniformly within the 137–143 mEq/L range, while blood urea nitrogen varied from 14 to 35 mg/dL and creatinine from 0.69 to 1.55 mg/dL. Transaminase levels showed ALT between 8 and 35 U/L and AST between 12 and 49 U/L. Albumin concentrations fell between 3.0 and 4.3 g/dL, total bilirubin between 0.2 and 0.6 mg/dL, and INR was consistently 1.0–1.2. Arterial blood gases demonstrated pH values from 7.30 to 7.43, PaCO₂ from 40 to 93 mmHg, and PaO₂ from 234 to 370 mmHg.

**Table 2:** Baseline Laboratory and Arterial Blood Gas Parameters

Case	Hemoglobin (g/dL)	WBC (1,000/mm3)	Platelets (1,000/mm3)	Sodium (mEq/L)	BUN (mg/dL)	Creatinine (mg/dL)	ALT (U/L)	AST (U/L)	Albumin (g/dL)	Total bilirubin (mg/dL)	INR	Arterial blood gas		
												pH	PaCO2 (mmHg)	PaO2 (mmHg)
1	7.9	10.6	373	138	14	1.03	8	15	4.0	0.2	1.2	7.30	42	329
2	7.5	7.9	192	142	29	0.70	23	29	3.0	0.3	1.0	7.33	78	266
3	8.4	4.0	119	143	24	0.91	35	23	4.0	0.3	1.0	7.41	60	247
4	10.7	2.9	186	143	35	1.39	9	16	4.3	0.5	1.0	7.37	40	356
5	10.8	6.2	326	141	18	1.55	13	49	3.4	0.3	1.0	7.43	45	370
6	10.2	14.0	388	143	22	1.25	21	12	3.3	0.3	1.0	7.35	53	234
7	7.3	2.7	165	137	16	0.69	8	25	4.0	0.6	1.0	7.37	93	266

BUN, blood urea nitrogen; ALT, alanine aminotransferase; AST, aspartate aminotransferase; INR, international normalized ratio.

### Donor and intraoperative characteristics

The seven donors ranged in age from 21 to 45 years; six were male and one female (Table 3). Causes of death included head trauma (*n* = 4), anoxia (*n* = 2), and stroke (*n* = 1). Operative times varied between 278 and 503 min, during which packed red blood cells transfused ranged from 140 to 2380 ml, fresh frozen plasma from 0 to 1820 ml, and platelets from 0 to 700 ml. Cold ischemic times spanned 413 to 811 min, and VA–ECMO support durations ranged from 128 to 277 min.

**Table 3:** Donor demographics and intraoperative outcomes

Case	Donor information			Intraoperative outcomes					
	Age	Gender	Cause of death	Operative time (mins)	Intra-op blood transfusion(ml)			Ischemic time (mins)	VA-ECMO time (mins)
					pRBC	FFP	Plt		
1	33	Male	Head trauma	700	280	0	700	445	277
2	21	Male	Anoxia	2380	1820	700	2380	413	243
3	39	Male	Anoxia	560	0	140	560	445	230
4	45	Male	Head trauma	280	140	0	280	427	223
5	28	Male	Head trauma	280	0	0	280	811	128
6	45	Female	Stroke	140	0	0	140	572	142
7	40	Male	Head trauma	1400	560	320	1400	685	145
pRBC, packed red blood cells; FFP, fresh frozen plasma; Plt, platelets; VA-ECMO, veno-arterial extracorporeal membrane oxygenation.									

BUN, blood urea nitrogen; ALT, alanine aminotransferase; AST, aspartate aminotransferase; INR, international normalized ratio.

### Postoperative complications and Follow-Up outcomes

Only one patient (Case 3) developed de novo donor-specific antibodies in the early postoperative period (Table 4). One patient had PGD grade 3 injury (Case3). 2 patients had AKI stage 3. Dialysis was required in the two patients with stage 3. No patient suffered cerebrovascular accident, bowel ischemia, or digital ischemia. Two recipients developed DVT, no PE occurred. One event arose in the upper extremity, and the other in the lower extremity with both above-knee and below-knee involvement. None of the DVTs were attributable to ECMO cannulation. No hemothorax was observed after lung transplantation. No post-transplant ECMO was necessary. Intensive care unit stays ranged from 2 to 58 days, and durations of mechanical ventilation varied between 1 and 58 days. Overall hospital stays spanned 10 to 130 days. Follow-up periods extended from 84 to 914 days, and at last follow-up all seven patients were alive without evidence of chronic lung allograft dysfunction or disease recurrence.

**Table 4:** Postoperative complications and follow-up outcomes

Case	de novo DSA	PGD grade	AKI stage	Dialysis	CVA	Bowel ischemia	Digital ischemia	DVT	PE	Hemothorax	post ope ECMO	post ope ECMO days	ICU stay (days)	Post LTx ventilator (days)	Hospital stay (days)	Follow-up period (days)	CLAD Status	Survival
1	No	0	1	No	No	No	No	No	No	No	No	0	10	7	16	914	NED	Live
2	No	1	1	No	No	No	No	Yes	No	No	No	0	24	5	130	766	NED	Live
3	Yes	1	0	No	No	No	No	No	No	No	No	0	4	2	38	483	NED	Live
4	No	1	1	No	No	No	No	No	No	No	No	0	2	1	10	469	NED	Live
5	No	1	3	Yes	No	No	No	No	No	No	No	0	58	58	59	356	NED	Live
6	No	3	3	Yes	No	No	No	Yes	No	No	No	0	14	1	18	84	NED	Live
7	No	1	1	No	No	No	No	No	No	No	No	0	7	2	11	199	NED	Live

de novo DSA, de novo donor-specific antibody; PGD, primary graft dysfunction; AKI, acute kidney injury; CVA, cerebrovascular accident; DVT, deep venous thrombosis; PE, pulmonary embolism; ECMO, extracorporeal membrane oxygenation; ICU, intensive care unit; LTx, lung transplantation; NED, no evidence of disease.

### Comparative context with contemporaneous central VA-ECMO cases

**Fig. 1:**
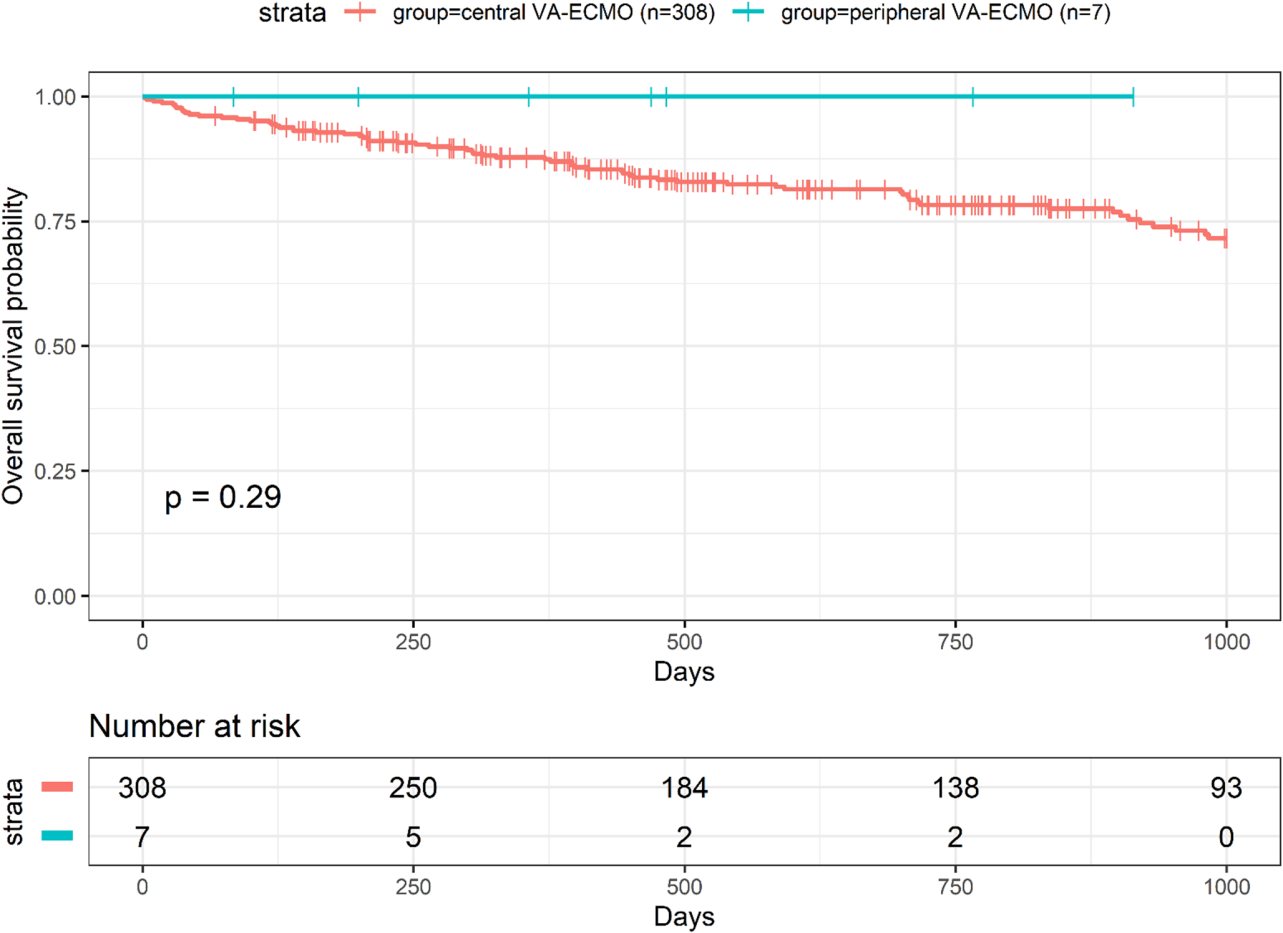
Kaplan–Meier overall survival stratified by intraoperative VA-ECMO (central vs. peripheral). Numbers at risk are displayed below; tick marks denote censoring. P value is from the log-rank test. VA-ECMO, veno-arterial extracorporeal membrane oxygenation

To contextualize these findings, we compared all lung transplants at our center supported intraoperatively with central VA-ECMO (*n* = 308) versus peripheral VA-ECMO (*n* = 7) (Table 5). Recipients in the peripheral cohort were younger. Pre-operative hemoglobin was lower (8.4 vs. 11.4 g/dL), while creatinine was modestly higher (1.0 vs. 0.8 mg/dL). Despite longer cold ischemic times (7.4 vs. 5.7 h) and numerically longer ECMO run time (3.7 vs. 2.8 h), intraoperative complication signals were low. As expected for technically demanding re-do procedures, pRBC transfusion during the case was higher in the peripheral cohort (560 [280–1400] vs. 280 [0–560] mL), whereas rates of FFP and platelet transfusion were similar. There was no significant diffrence in complication. The need for post-operative ECMO was 0% in the peripheral cohort versus 17.9% in the central group. ICU stay, duration of mechanical ventilation, and overall hospital length of stay were broadly comparable across cohorts. Overall survival through follow-up is illustrated in the Kaplan–Meier analysis (Fig. 1); all seven peripheral-ECMO patients were alive at last follow-up, and the curve did not suggest inferiority versus central VA-ECMO.

**Table 5:** Patient characteristics according to intraoperative central or peripheral VA-ECMO use

Variable	central VA- ECMO(*n* = 308)	peripheral VA-ECMO (*n* = 7)	*P* value
*Pre-operative Characteristics*			
Age, years	61.0 [50.0–66.0]	42.0 [38.0–55.0]	0.01
Gender			0.13
Male	164 (53.2%)	6 (85.7%)	
Female	144 (46.8%)	1 (14.3%)	
BMI, kg/m2	26.5 [21.9–29.5]	22.7 [20.6–24.0]	0.12
BSA, m2	1.9 [1.7-2.0]	1.8 [1.7–1.9]	0.43
Single/Bilateral			0.60
Single	44 (14.3%)	0 (0.0%)	
Bilateral	264 (85.7%)	7 (100.0%)	
Smoking history	141 (45.8%)	1 (14.3%)	0.13
Hypertension	169 (54.9%)	4 (57.1%)	1.00
Diabetes	88 (28.6%)	3 (42.9%)	0.42
CKD	24 (7.8%)	2 (28.6%)	0.11
Etiology			< 0.001
CLAD	0 (0.0%)	7 (100.0%)	
ILD	148 (48.1%)	0 (0.0%)	
COPD	36 (11.7%)	0 (0.0%)	
PAH	29 (9.4%)	0 (0.0%)	
ARDS	36 (11.7%)	0 (0.0%)	
Others	59 (19.2%)	0 (0.0%)	
Laboratory			
Hemoglobin, g/dL	11.4 [9.3–13.2]	8.4 [7.5–10.7]	0.01
WBC, 1,000/mm3	9.2 [7.1–11.8]	6.2 [2.9–10.6]	0.05
Platelets, 1,000/mm3	238.0 [178.0-304.0]	192.0 [165.0-373.0]	0.99
Sodium, mEq/L	139.0 [137.0-141.0]	142.0 [138.0-143.0]	0.10
Creatinine, mg/dL	0.8 [0.6–0.9]	1.0 [0.7–1.4]	0.02
ALT, U/L	17.0 [11.5–26.5]	13.0 [8.0–23.0]	0.40
AST, U/L	21.0 [17.0–28.0]	23.0 [15.0–29.0]	0.81
Albumin, g/dL	3.9 [3.5–4.2]	4.0 [3.3-4.0]	0.48
Total bilirubin, mg/dL	0.5 [0.4–0.8]	0.3 [0.3–0.5]	0.02
*Donor inforomation*			
Age, years	35.0 [26.0–45.0]	39.0 [28.0–45.0]	0.86
Gender			0.43
Male	202 (65.6%)	6 (85.7%)	
Female	106 (34.4%)	1 (14.3%)	
Cause of death			0.54
Anoxia	123 (39.9%)	2 (28.6%)	
Stroke	80 (26.0%)	1 (14.3%)	
Head Trauma	101 (32.8%)	4 (57.1%)	
Other	4 (1.3%)	0 (0.0%)	
*Intra-operative outcomes*			
Operative time (hours)	6.1 [5.1–7.9]	6.8 [6.0-7.9]	0.39
Intra-op blood transfusion; pRBC	280.0 [0.0-560.0]	560.0 [280.0-1400.0]	0.04
Intra-op blood transfusion; FFP	0.0 [0.0-140.0]	140.0 [0.0-560.0]	0.12
Intra-op blood transfusion; Plt	0.0 [0.0-140.0]	0.0 [0.0-280.0]	0.33
Ischemic time (hours)	5.7 [4.9–6.8]	7.4 [7.1–11.4]	0.002
VA ECMO time (hours)	2.8 [2.3–3.3]	3.7 [2.4-4.0]	0.36
*Post-operative outcomes*			
de novo DSA	50 (16.2%)	1 (14.3%)	1.00
PGD grade3	57 (18.5%)	1 (14.3%)	1.00
AKI	161 (52.3%)	6 (85.7%)	0.13
Dialysis	50 (16.2%)	2 (28.6%)	0.33
CVA	10 (3.2%)	0 (0.0%)	1.00
Bowel Ischemia	3 (1.0%)	0 (0.0%)	1.00
Digital Ischemia	9 (2.9%)	0 (0.0%)	1.00
DVT	151 (49.0%)	2 (28.6%)	0.45
PE	36 (11.7%)	0 (0.0%)	1.00
Hemothorax	81 (26.3%)	0 (0.0%)	0.20
post ECMO use	55 (17.9%)	0 (0.0%)	0.61
ICU stay	9.0 [5.0-20.5]	10.0 [4.0–24.0]	1.00
Post transplant ventilator	2.0 [1.0-0.5]	2.0 [1.0–7.0]	0.75
Hospital stay	21.0 [13.0–37.0]	18.0 [11.0–9.0]	0.82
Follo-up period (days)	700.0 [330.5-1221.5]	469.0 [199.0-766.0]	0.16

Continuous data are shown as medians and and interquartile ranges (Q1-Q3) for days. CLAD, chronic lung allograft dysfunction; AKI, acute kidney injury; BMI, body mass index; BSA, body surface area; ECMO, extracorporeal membrane oxygenation; LAS, lung allocation score; COPD, chronic obstructive pulmonary disease; CPFE, combined pulmonary fibrosis and emphysema; ILD, interstitial lung disease; ARDS, acute respiratory distress syndome; COVID-19, coronavirus disease 2019; PAH, pulmonary arterial hypertension; WBC, white blood cell; BUN, blood urea nitrogen; INR, international normalized ratio; PRA, panel reactive antibody

## Discussion

This study aimed to evaluate the feasibility and safety of using full dose anticoagulation-free peripheral VA-ECMO in lung transplantation. The findings suggest that peripheral VA-ECMO with reduced-dose heparin is a viable strategy, yielding favorable clinical outcomes—no deaths during follow-up, preserved graft function, and low postoperative complication rates. Importantly, there were no instances of PE or limb ischemia. This supports the hypothesis that full dose heparin-free peripheral VA-ECMO can be employed safely without increasing the risk of major thromboembolic complications. This study contributes novel insights into the use of full dose heparin-free peripheral VA-ECMO in lung transplantation. The key strength of this study lies in its single-center, retrospective design, which allowed for a consistent protocol and patient cohort, minimizing confounding variables often seen in multicenter studies. The absence of limb ischemia and　PE supports the safe implementation of peripheral VA-ECMO without exposing patients to anticoagulation-related risks. We preferentially used a 16-Fr femoral arterial cannula, which likely mitigated distal malperfusion while preserving adequate flows; notably, no distal limb perfusion complications occurred despite full dose heparin-free management. Our study also highlights that clinical outcomes such as survival rates, pulmonary complications, and graft function were comparable to those observed in patients with central VA-ECMO [10–12]. These results could provide an argument for the broader use of peripheral VA-ECMO, particularly in lung transplant recipients who may benefit from a less invasive and potentially safer strategy. Previous studies have shown that central VA-ECMO is an effective tool for providing circulatory support during lung transplantation via clamshell incision [10–12].

Previous study showed that re-do lung transplant recipients required a median of 1,600 mL (IQR 800–2,000 mL) of red blood cell transfusion, whereas in our full dose heparin-free peripheral VA-ECMO–supported cohort for re-lung transplant the median requirement was only 560ml [21]. Although direct inter-facility comparison is limited, these data suggest that our protocol may substantially reduce transfusion needs—consistent with other reports of full dose heparin-free ECMO strategies demonstrating decreased bleeding and transfusion requirements without compromising clinical outcomes [13, 22]. Previously, we published a case report of Veno-arterial extracorporeal membrane oxygenation without therapeutic anticoagulation for intra-operative cardiopulmonary support during lung transplantation [22], which demonstrated the safety of VA-ECMO without full anticoagulation. In our practice, we administer 5,000 U of unfractionated heparin before central VA-ECMO cannulation. Patients were not monitored with bleeding parameters such as ACT or aPTT during lung transplant procedure. To avoid thrombotic complication in the ECMO circuit, flow should be maintained at least 2.5 L/min during lung transplant procedure. If there is consistent reduction in circuit flow due to hypovolemia or peripheral vasodilation, repeat administering unfractionated heparin to reduce thrombogenicity is recommended. In addition, we recently reported the feasibility of VV-ECMO without systemic anticoagulation for respiratory failure patients [13], which has been shown to decrease blood transfusions and also reduce the number of required oxygenator exchanges. Furthermore, in both reports mentioned above, we made use of heparin-coated tubing and oxygenators to reduce bleeding and adverse thrombotic events. We believe this strategy is important for safely using VA or VV-ECMO without systemic anticoagulation; however, full dose heparin free peripheral VA-ECMO may increase the risk of thrombotic complications—such as circuit thrombosis, limb ischemia, and embolic stroke—which necessitates close monitoring (regular Doppler ultrasound and limb perfusion assessment) and the use of heparin-coated circuits or localized anticoagulation strategies when appropriate. Recently, innovations in ECMO circuit technology have decreased the pro-coagulant nature of ECMO. These include the use of heparin-coated tubing, centrifugal pumps and polymethylpentene oxygenators [6, 8, 9]. The advent of these new techniques has played a large role in being able to implement extracorporeal support without the use of systemic anticoagulation.

This study distinguishes itself by focusing specifically on peripheral VA-ECMO, which has been less widely studied. Unlike central VA-ECMO, peripheral VA-ECMO involves femoral venous and arterial cannulation, a technique that has been associated with easier insertion for re-do lung transplant or sparing sternum, making it particularly appealing for patients who may be at higher risk for bleeding or complications due to other comorbidities. Our findings that there were no PE complications in the peripheral VA-ECMO cohort suggest that this approach can be as safe as traditional central ECMO, if not more so, in reducing the potential for such adverse outcomes. Peripheral VA-ECMO can be performed with a smaller cannulation size and reduced risk of major vessel injury such as ascending aorta dissection, thus minimizing complications that could occur with central ECMO. This study also provides evidence that full dose heparin-free ECMO may help to address the growing concern of blood product use in high-risk populations. By reducing the need for anticoagulation, this strategy minimizes the risk of bleeding, which is often a major contributor to postoperative complications, prolonged ICU stays, and increased mortality in lung transplant recipients. Moreover, fewer transfusions are associated with improved kidney function, as excessive blood product transfusions can contribute to AKI in transplant patients. In our seven-case series, AKI occurred in 6 of 7 patients (86%), yet only 2 of 7 (29%) required dialysis, suggesting this approach may mitigate the progression to renal failure. These hypothesis-generating findings warrant confirmation in larger cohorts.

The safety of heparin-free ECMO is time-dependent and relates to cumulative thrombotic risk within the circuit, at cannula tips, and in native vessels. In our series, heparin avoidance was confined to short, intraoperative peripheral VA-ECMO runs (128–277 min), during which we observed no circuit thrombosis, embolic events, limb ischemia, circuit exchange, or low-flow instability. In lung transplantation, it is uncommon for operative time to exceed 24 h; within this window, we believe intraoperative peripheral VA-ECMO can be used safely without systemic heparin when adequate flows are preserved and surgical hemostasis is prioritized. Heparin-free VV-ECMO has been reported with no excess major thrombotic complications versus anticoagulated care (Kurihara et al., 2020), and VA-ECMO without routine systemic anticoagulation in the ICU setting was associated with fewer overall adverse events and lower blood product utilization without increased thrombosis or mortality (Wood et al., 2020). Taken together, these observations suggest that heparin-free support is reasonable for short intraoperative runs under vigilant monitoring, while acknowledging that patients with prothrombotic states (e.g., HIT), intracardiac stasis/poor pulsatility, or non–heparin-coated circuits should not be managed with a fully heparin-free approach and warrant earlier transition to systemic anticoagulation.

Based on these data, our institution favors a limited-application policy rather than universal use. Specifically, heparin-free intraoperative peripheral VA-ECMO will be considered for re-do lung transplant candidates in whom bleeding risk is expected to predominate. Candidate indications include (i) anticipated diffuse adhesion-related bleeding (e.g., re-do cases with dense pleural or perihilar adhesions, prior intrapericardial cardiac procedures, or prior thoracic radiation/operations), (ii) pre-existing high bleeding risk (active antiplatelet therapy that cannot be interrupted, known coagulopathy or platelet dysfunction), and (iii) a history of recurrent major bleeding on systemic anticoagulation. Exclusions/relative contraindications include prothrombotic states (e.g., heparin-induced thrombocytopenia), cardiac failure with intracardiac stasis, inability to sustain circuit flows ≥ 2.5 L/min after correction of reversible factors, non–heparin-bonded circuits and anticipated prolonged support beyond the intraoperative period.

Despite the promising findings, this study has several limitations. First, the retrospective design introduces inherent biases, such as selection bias, which could affect the generalizability of the results. In addition, our cohort did not include patients with reduced left ventricular systolic function; therefore, the safety of full dose heparin-free peripheral VA-ECMO may not generalize to populations with low EF, in whom the risk of LV distension or thrombus formation may be higher when anticoagulation is minimized.　Additionally, while the sample size was sufficient for initial conclusions, larger, multicenter studies are needed to further validate the safety and effectiveness of full dose heparin-free peripheral VA-ECMO in a broader patient population. Another limitation is the absence of long-term follow-up data regarding chronic rejection, graft survival, and other late complications, which would provide a more comprehensive understanding of the long-term impact of this approach. Additionally, the study was conducted at a single center, and results may vary in institutions with different protocols, patient demographics, or institutional expertise.

## Conclusion

In this single-center cohort, full dose heparin-free peripheral VA-ECMO during lung transplantation was feasible and appeared safe: we observed no deaths during follow-up, preserved graft function, and low rates of thrombotic complications, including limb ischemia. Although a contemporaneous central VA-ECMO control was not included and direct equivalence cannot be inferred, our outcomes are consistent with previously reported series. These findings support the selective use of peripheral VA-ECMO with reduced anticoagulation in lung transplantation; confirmation in prospective, multicenter studies is warranted.

## Data Availability

The datasets generated and/or analyzed during the current study are available from the corresponding author on reasonable request.
